# Cytokine interactions and chemokine dysregulations in mastitis immunopathogenesis: insights from transcriptomic profiling of milk somatic cells in tropical Sahiwal (*Bos indicus*) cows

**DOI:** 10.3389/fimmu.2025.1554341

**Published:** 2025-03-24

**Authors:** Lija Satheesan, Ajay Kumar Dang, Rani Alex

**Affiliations:** ^1^ Lactation and Immuno-Physiology Laboratory, Animal Physiology Division, Indian Council of Agricultural Research (ICAR)-National Dairy Research Institute, Karnal, Haryana, India; ^2^ Division of Animal Genetics and Breeding, Indian Council of Agricultural Research (ICAR)-National Dairy Research Institute, Karnal, Haryana, India

**Keywords:** bovine mastitis, transcriptomics, differential expressed genes, cytokine interactions, chemokine markers, mRNA sequencing

## Abstract

**Introduction:**

Bovine mastitis causes a significant loss to the dairy industry by affecting the quantity and quality of milk. Addressing this challenge, the present study will leverage advanced omics techniques for early mastitis detection in early lactating Sahiwal cows (*Bos indicus*). This was the first differential transcriptomic study investigating the alterations in gene expression in milk somatic cells during the progression of naturally occurring mastitis in indigenous Sahiwal cows.

**Methods:**

Cows were grouped into healthy (H), subclinical mastitis (SCM) and clinical mastitis (CM) groups by thoroughly screening them using the California Mastitis Test (CMT) and milk somatic cell counts (SCC). This was followed by detailed milk composition analysis, differential leukocyte counts (DLC), and microbiological culture.

**Results:**

The differential gene expression of milk SCs through transcriptome profiling identified 83 and 76, up-regulated and 157 and 192 down-regulated genes in CM vs H and SCM vs H groups (log2 fold change ≥1 and ≤-1, p < 0.05) respectively. Pathway analysis revealed that upregulated genes were enriched in pathways such as phagosome activity, IL-17 signalling, Th1 and Th2 cell differentiation, while downregulated genes were linked to RIG-I-like receptor signalling, NK cell cytotoxicity, and Toll-like receptor signalling and Cytokine-cytokine receptor interactions. Notably, the study underscores the roles of chemokines CCL8, CCL2, and CXCL10 in immune cell recruitment during mastitis, where their downregulation suggests impaired mammary immune defense that governs Chemokine signalling pathways. Further, the comparative analysis with the previously available milk SCs proteome data identified the downregulation of chemokines signalling pathways during mastitis.

**Discussion:**

Overall, this research enhances our understanding of mastitis pathogenesis and emphasizes that these targeted chemokines may boost mammary resilience through immunomodulation, genetic selection and genome editing or by utilising adjuvants in vaccine development that restore chemokine signalling offers a potential strategy to improve mastitis resistance in dairy cattle.

## Introduction

Bovine mastitis continues to be a critical production disease, resulting in significant economic losses for the dairy sector by negatively impacting both milk yield and quality (1). Mastitis manifests in two forms: subclinical and clinical. The subclinical form is particularly challenging because it lacks visible symptoms but typically triggers an increase in immune cell influx to the udder, resulting in elevated milk somatic cell counts (SCCs). Sahiwal cows (*Bos indicus*), as a premier dairy breed in tropical regions of India and across the globe in countries like Australia and parts of Africa, exhibit milk SCC levels that can range from 2–5 to over 5×10⁵ cells/mL, reflecting the transition of mastitis from subclinical to clinical stages (2). An elevated SCC serves as a key indicator of mastitis, which can be detected using conventional methods such as the California mastitis test (CMT) or automated somatic cell counters. While SCC provides a broad assessment of udder health, differential leukocyte count (DLC) in milk offers a more refined measure of the inflammatory response, allowing for earlier and more precise detection. Consequently, milk somatic cells (SCs) are essential indicators for early mastitis detection in cattle, as they play a vital role in the immune defence of the mammary gland against natural infections (2, 3). However, microbiological culture is essential for confirming the presence of mastitis-causing pathogens, offering insights into the etiological agents involved.

Gaining insights into the molecular and immune responses of the host during natural mastitis is crucial. Emerging research suggests that the host’s response at early stages, including cytokine and chemokine production, significantly influences mastitis regulation (4). Advances in next-generation sequencing (NGS) have revolutionized transcriptomics, enabling large-scale RNA sequencing (RNA-seq) to quantify gene expression, identify isoforms, and discover novel transcripts (5). High-throughput RNA-seq captures numerous transcripts via cDNA sequencing, which has transformed the study of livestock transcriptomes, allowing for detailed analysis of differentially expressed genes and biological pathways (6, 7).

Understanding the genetic and molecular mechanisms that underlie the pathogenesis of naturally occurring bovine mastitis is fundamental for discovering new targets for treatment and vaccination in a herd (8). However, there is limited research on milk somatic cell transcriptomic analysis during mastitis using advanced RNA sequencing and bioinformatics tools, particularly in indigenous cattle breeds (9). To date, no comparative transcriptomic profiling of milk somatic cells from healthy, subclinical, and clinical mastitis-affected Sahiwal cows has been documented.

Milk SC protein expression in healthy and mastitis cows offers insights into dairy applications and mammary immune function (10). Further, the comparative analysis with the previously available milk SC proteome data identified the downregulation of chemokine signalling pathways during mastitis (3). This study, therefore, seeks to identify differentially expressed genes (DEGs) in milk somatic cells from naturally infected states of mastitis infection in Sahiwal cows for the first time. Additionally, it aims to expand our understanding of host cytokine interactions and chemokine dysregulations during immunopathogenesis of mastitis, which may serve as potential targets for therapeutic interventions and vaccine development to combat bovine mastitis.

## Materials and methods

### Chemicals and plasticware

All chemicals, reagents, and plasticware were procured from Thermo Fisher Scientific (Waltham, MA, USA) unless stated otherwise.

### Ethics statement

The approval of all the experiments carried out in this research work was obtained from the Animal Ethics Committee of the National Dairy Research Institute, Karnal, according to the Committee for Control and Supervision of Experiments on Animals rules, laid down by the Government of India, No. 1705/GO/Re/L/13/CPCSEA dt.23/07/2021.

### Study area, screening of cows, and collection of milk samples

The current study was conducted at the Livestock Research Centre, Karnal, India. The Institute is situated at coordinates 29°43′14.4″ N latitude, 76°58′55.2″ E longitude, with an altitude of 250 m above the mean sea level in the bed of Indo-Gangetic alluvial plain. The climate is hot (45°C) in the summer and chilly (4°C) in the winter, as the area is tropical. Annual rainfall is ~70 cm, and relative humidity varies from 41% to 85%. A semi-intensive system of rearing was followed for the cows on the farm, where the cows were housed in a well-ventilated shed with provisions for individual standing/resting space, feeding manger, and watering troughs as per Bureau of Indian Standards (BIS)s. All the cows selected (n = 71) were non-pregnant, in their days in milk ranges from 70 to 98, and a milking twice a day (morning and evening) system was practised. These cows were multiparous (parity 3 to 5) with an average body condition score of 3.5, fed with *ad libitum* green fodder and a concentrate diet (20% crude protein and 70% total digestible nutrient) as per practices followed in the station for lactating cows.

In the present study, the mid-stream quarter milk samples were collected during morning milking time (6.30 a.m.); 100 mL of mid-stream milk samples was collected aseptically from the quarter in a sterile centrifuge tube, as per National Mastitis Council guidelines, and immediately transported to laboratories for further analysis. CMT scoring was conducted for initial screening. Briefly, 2 mL of fresh foremilk from each quarter was added to the corresponding chamber of a CMT plastic paddle and mixed with an equal volume (2 mL) of CMT reagent at room temperature. The paddle was gently swirled in a circular motion to facilitate mixing. An increase in viscosity indicated a rise in quarter SCC, and the CMT reaction was visually assessed by a single investigator 45 seconds after reagent addition. Furthermore, the milk samples were collected for the milk SCC, DLC, and microbiological culture test and then further retrospectively grouped as healthy, subclinical, and clinical mastitis cows. The cows were grouped as healthy (H) if they had no previous history of clinical mastitis, CMT score 0 (negative) and low milk SCC < 2 × 10^5^ cells/mL. Cows with milk SCC 2–5 × 10^5^ cells/mL, without clinical signs of mastitis, and with CMT score 1 (slight gel formation) were included in the subclinical mastitis (SCM) group. Cows that exhibited the early stages of udder inflammation, flakes of size 1–2 mm in milk (5–10/mL), and CMT score 2 (distinct gel formation) and were categorized into the clinical mastitis (CM) group (advanced clinical mastitis cases were excluded, as antibiotic and anti-inflammatory treatments were started in such cows) (11). Based on CMT scoring, milk SCC, and bacteriological culture, the culture-negative milk samples with CMT score 0 and SCC < 2 × 10^5^ cells/mL were retrospectively classified as H (n = 18). Similarly, culture-positive samples with CMT score 1 and SCCs of 2–5 × 10^5^ cells/mL, and CMT score 2 and SCC >5 × 10^5^ cells/mL were grouped in the SCM (n = 18) and CM (n = 18) groups, respectively.

### Milk somatic cell count analysis

Milk SCC was estimated using the Lactoscan milk SCC counter (Milkotronic Ltd., Stara Zagora, Bulgaria). The Lactoscan somatic cell counter is based on fluorescent image cytometry for counting cells. Briefly, 100 µL of fresh milk was mixed with Sofia Green Lyophilized dye in a microtube, and 8 µL was pipetted onto lactochip and loaded in the machine. The system of Lactoscan focuses automatically on the chip. The algorithm of analysis of digital images determines the number of fluorescent cells and counts their concentration (12).

### Milk composition analysis

The milk composition analysis for the fat (%), solid non-fat (SNF %), protein (%), lactose (%), electrical conductivity (EC) (mS/cm) and pH were determined automatically using a Lactoscan MCC Combo (Milkotronic Ltd., Stara Zagora, Bulgaria) for the collected milk samples (13).

### Milk differential leukocyte count analysis

DLC for milk samples was conducted by preparing methylene blue-stained smears to quantify neutrophils, macrophages, lymphocytes, and epithelial cells in each sample. Grease-free glass slides were marked, and 10 µL of milk was spread over a 1-cm^2^ area using a spreader. The smear was air-dried, fixed with 95% ethyl alcohol for 3 minutes, defatted with xylene for 12 minutes, and rinsed with 60% ethyl alcohol. It was stained with methylene blue for 15 minutes, rinsed, and air-dried. The smear was examined at ×100 under an Olympus IX51 microscope (Olympus, Tokyo, Japan). The percentage of various milk SCs was calculated using the formula below (12, 13).


$$\text{DLC of a particular cell type }\left(\%\right)=\frac{\text{No}.\text{ of that particular cell}}{\text{Total no}.\text{ of cells}}\times 100.$$

### Milk microbiological analysis for mastitis pathogens

Milk samples were analysed for the microbiological examination according to ISO 4833-2:2013/Amd 1:2022. The quarter milk samples were analysed for probable mastitis-causing pathogens on selective media agar by pour plate method using different selective agar plates, namely, Baird-Parker agar, blood agar, and Eosin Methylene Blue (EMB) agar. Nutrient agar (28.0 g/L) was dissolved in distilled water, boiled, sterilized at 120°C for 15 minutes, and cooled before being poured into sterile petri plates. Similarly, EMB agar (35.96 g/L), BPA agar (63 g/950 mL) and blood agar base (40.5 g/L) were prepared, sterilized, and poured. Bacterial colonies from the nutrient agar were picked up by inoculation loop and subcultured by streaking on a different selective agar plate. After incubation, the plates were examined for the growth of various bacteria on different selective agar plates. At the end of incubation (37°C for 24–48 h), the plates were observed for the appearance of typical colonies. The characterization of the isolates was conducted by Gram staining. Further, IMViC (Indole, Methyl Red, Voges Proskauer, and Citrate) test and catalase test were performed. The IMViC test helps differentiate coliform bacteria by assessing their metabolic capabilities, such as indole production, acid fermentation, acetoin production, and citrate utilization. The catalase test detects the presence of the catalase enzyme, which breaks down hydrogen peroxide into water and oxygen, distinguishing *Staphylococcus* from *Streptococcus*. These tests are essential for identifying mastitis-causing pathogens in milk samples (12).

### Isolation of milk somatic cells

The collected quarter milk samples were filtered through a 40-µm nylon filter into 50-mL siliconized tubes and centrifuged at 3,000 rpm for 15 minutes at 4°C. After removing the fat layer and discarding the supernatant, the cell pellet was resuspended in 1 mL 1× Dulbecco's Phosphate-Buffered Saline (DPBS) (pH 7.2) and layered over 30% Percoll for gradient centrifugation at 3,000 rpm for 30 minutes to purify milk SCs by removing bacteria and contaminants. The top layer was collected, washed twice with DPBS at 4°C, 3,000 rpm for 8 minutes, and suspended in 1 mL 1× DPBS in 2-mL autoclaved Eppendorf tubes. The cells were then centrifuged at 4°C and 10,000 rpm for 5 minutes, and the final cell pellet was stored at −80°C for further analysis (2, 3).

### Sample selection for transcriptomic analysis

After microbiological analysis, the isolated milk somatic cell samples based on the criteria that milk samples that tested negative on cultures and no previous history of mastitis, low milk SCC (<2 × 10⁵ cells/mL) and milk CMT score 0 were classified retrospectively as the H (n = 18) group. Cows without clinical signs of mastitis and having milk SCC (2–5 × 10⁵ cells/mL) and CMT score of 1 (slight gel formation) with culture-positive milk samples were classified as the SCM (n = 18) group. Cows that exhibited the early stages of clinical signs of mastitis, such as mild udder inflammation changes, along with the presence of flakes of size 1–2 mm (5-10/mL) in the milk, SCC (>5 × 10⁵ cells/mL) and a CMT score of 2 (indicating distinct gel formation) and are culture-positive were classified as the clinical mastitis CM (n = 18) group (3, 11).

### RNA isolation

To isolate total RNA, 1 mL of TRI Reagent^®^ was added to a 1 × 10^6^ cell pellet in a 1.5-mL Eppendorf tube, followed by cell lysis at room temperature. The samples were stored at −20°C until use. After thawing, 200 µL of chloroform was added, and the tubes were vigorously shaken, incubated for 5 minutes at room temperature, and centrifuged at 12,000 *g* for 15 minutes at 4°C. The RNA-containing upper phase was carefully transferred to a fresh tube, mixed with 500 µL of chilled isopropanol, and incubated for 10 minutes to precipitate the RNA. Centrifugation at 12,000 *g* for 10 minutes at 4°C yielded the RNA pellet, which was washed with 75% ethanol and centrifuged twice at 7,500 *g* for 5 minutes at 4°C. After removing the residual ethanol, the RNA pellet was air-dried and dissolved in 20 µL of nuclease-free water. The RNA was further purified using the Qiagen RNeasy kit, and its yield and purity were assessed by NanoDrop™ 1000 spectrophotometer (Thermo Fisher Scientific, USA) and TapeStation (Agilent, Santa Clara, CA, USA), ensuring RNA integrity (RIN ≥ 7.0). The final concentration was measured using the Qubit RNA HS assay kit (Q32855), and samples were stored at −80°C for RNA sequencing.

### RNA sequencing

RNA sequencing libraries were constructed using the Illumina-compatible NEBNext^®^ Ultra™ II Directional RNA Library Prep Kit (New England Biolabs, Ipswich, MA, USA) for mRNA transcript analysis of nine biological replicates from the H, SCM, and CM samples. Briefly, 100 ng of total RNA was isolated, fragmented, and primed from each sample, followed by first- and second-strand cDNA synthesis. The double-stranded cDNA was purified, end-repaired, adenylated, and ligated to Illumina adapters, with the second strand excised using USER enzyme. Adapter-ligated cDNA was purified and underwent 14 cycles of PCR for indexing and enrichment. The final sequencing library was purified, quality checked, and quantified using a Qubit fluorometer (Thermo Fisher Scientific, MA, USA), and fragment size distribution was analysed on an Agilent 2200 TapeStation. The libraries were then paired-end sequenced on an Illumina NovaSeq X Plus sequencer (Illumina, San Diego, CA, USA) for 150 cycles.

### Raw data processing

Transcriptomic analysis began with raw data processing, where reads were assessed for quality using FastQC v.0.11.9 (14), and adapter sequences and low-quality bases (<q30) were removed using Trim Galore v. 0.4.0. The pre-processed high-quality reads were then aligned to the *Bos* reference genome (GCF_002263795.2_ARS-UCD1.3) from the NCBI database (https://www.ncbi.nlm.nih.gov/datasets/genome/GCF_002263795.2/) using HISAT2 v2.0.5 (15). Reads were classified as aligned or unaligned based on their match to the reference genome. Following alignment, expression analysis was performed at the transcript level, and the resulting data were used for downstream analyses such as Gene Ontology annotation and pathway analysis to determine the functional roles of each expressed transcript.

### Differential expression analysis

Transcript abundance was estimated using the FeatureCounts tool (v1.5.2), which provided absolute counts for each transcript (16). For differential expression analysis, DESeq2 (v1.42.1) was employed to identify differentially expressed transcripts (17). DESeq2 performs internal normalization by calculating the geometric mean for each transcript across all samples and fits negative binomial generalized linear models for each transcript using the Wald test for significance testing. The expressed transcripts were then categorized as upregulated, downregulated, or neutrally regulated based on a Log_2_ fold change cut-off of ±1 for subsequent functional analysis.

### Functional annotation and pathway analysis of identified DEGs

Transcripts were functionally annotated based on homology using the Basic Local Alignment Search Tool (BLAST) tool against Bovidae sequences from the UniProt database. Transcripts were assigned homologous proteins from other organisms if a match was found with an e-value less than e−5 and a minimum similarity of over 30%. For functional analysis, genes expressed commonly or uniquely in the H, SCM, and CM groups were represented using Venn diagrams created using the R package Venn Diagram (v.1.7.3) (18). Volcano plots were generated using the R package ggplot2 (v.3.4.4) (19), and heatmaps were produced using complexHeatmap (v.2.18.0) in R (v.4.3.2) (20). A gene interaction network was constructed for identifying hub genes with the highest degree of interactions using the cytoHubba plugin of Cytoscape (v.3.10.1) (21, 22). Additionally, the Database for Annotation, Visualization, and Integrated Discovery (DAVID) was used for the functional analysis of differentially expressed genes, and pathway analysis of DEGs and differentially expressed proteins (DEPs) identified from the previous proteomic data PXD045487 (3) was performed using the Kyoto Encyclopedia of Genes and Genomes (KEGG) (23).

### Validation of differential gene expression by quantitative real-time PCR

The differential expression observed by RNA-seq was validated through quantitative real-time PCR (qRT-PCR) for genes significant in immune responses, including *CCR7*, *CCL17*, *CCL22*, *CXCL10*, *CCL8*, and *CCL2*. Primers for qRT-PCR were designed using the Primer3 software (24), with details provided in **Supplementary Table 1**. cDNA was synthesized from stored RNA samples of the H, SCM, and CM groups, with RNA purity indicated by an OD_260_/OD_280_ ratio ranging from 1.8 to 2.0. RNA was reverse transcribed into cDNA using the RevertAid First Strand cDNA Synthesis Kit (Catalogue No. K1622, Thermo Fisher Scientific, USA) following the manufacturer’s protocol, with the RT reaction conducted in a thermal cycler (Bio-Rad, Hercules, CA, USA). The PCR assay was conducted in a 10-µL reaction volume containing Maxima SYBR Green/ROX QPCR Master Mix (2×) (Thermo Fisher Scientific), 0.2 µM of each primer, and 1 µL of cDNA, subjected to relative quantification via RT-qPCR. The concentration of each amplified cDNA sample was calculated relative to the endogenous controls GAPDH and β-actin.

### Statistical analysis

Data on SCC, composition, and DLC were analysed using analysis of variance (ANOVA) to compare groups at a 95% confidence level (p < 0.05). Differences in bacteriological colony counts between subclinical and clinical mastitis samples were assessed with an independent t-test. All statistical analyses were conducted using R (v.4.3.2) (25). For quantitative analysis, mRNA transcript abundance data were Log_2_-transformed and normalized. Genes quantified in at least two out of three replicates per group were filtered; the remaining values were imputed, and t-tests were applied. Significant differential abundance was determined based on a combination of Log_2_ fold change cut-offs (≤−1 and ≥1), p-value <0.05.

## Results

### Milk SCC, composition, and DLC of milk samples

**Figure 1:**
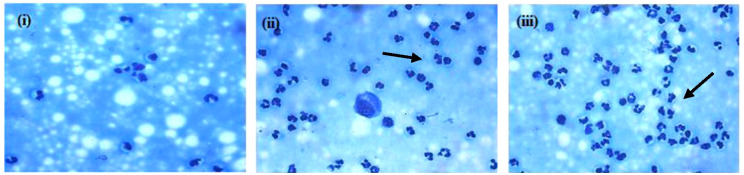
DLC of milk samples. (i) H, (ii) SCM, and (iii) CM on methylene blue staining, with arrows showing a high influx of neutrophils in mastitis condition compared to healthy samples (×1,000). DLC, differential leukocyte counts; H, healthy; SCM, subclinical mastitis; CM, clinical mastitis.

A significant increase in milk SCC (F = 192.52, p = 0.00) was observed in mastitis cows when compared to healthy cows. No significant difference was observed between the healthy, SCM, and CM groups on milk composition (%) such as fat (F = 1.68, p = 0.21), SNF (F = 1.29, p = 0.28), protein (F = 1.11, p = 0.34), and lactose (F = 0.66, p = 0.52). In contrast, the chemical properties like conductivity (F = 31.740, p = 0.00) and pH (F = 29.67, p = 0.00) showed significant differences between the H and CM groups. Among the healthy samples, milk DLCs revealed a prevalence of macrophages, followed by neutrophils, lymphocytes, and epithelial cells. Conversely, neutrophils were the predominant leukocyte type in the mastitis groups, followed by macrophages, epithelial cells, and lymphocytes. The mean ± SE values for SCC, nutritional and chemical compositional characteristics of milk samples with DLC, and neutrophil:macrophage (N:M) ratio of the healthy, subclinical, and clinical mastitis groups obtained are presented (**Table 1**). Methylene blue staining further highlighted the influx of neutrophils in milk during mastitis (**Figure 1**).

**Table 1:** Somatic cell count, milk composition, DLC, N:M ratio, and microbiological findings of the samples isolated from quarter milk samples of H, SCM, and CM groups of cows.

Milk sample parameters	Healthy	SCM	CM
Milk SCC (×10^5^ cells/mL)	1.20 ± 7.4^a^	3.56 ± 16.23^b^	7.60 ± 31.71^c^
Fat %	4.80 ± 0.32	4.4 ± 0.19	4.08 ± 0.31
SNF %	8.21 ± 0.18	8.41 ± 0.06	8.14 ± 0.07
Protein %	2.98 ± 0.07	3.07 ± 0.03	2.98 ± 0.03
Lactose %	4.48 ± 0.10	4.56 ± 0.03	4.46 ± 0.04
Conductivity (mS/cm)	5.57 ± 0.13^a^	5.95 ± 0.16^a^	7.55 ± 0.25^b^
pH	6.94 ± 0.02^a^	7.00 ± 0.02^a^	7.15 ± 0.02^b^
Neutrophils %	19.22 ± 0.57^a^	68.22 ± 1.32^b^	80.55 ± 0.68^c^
Macrophages %	70.88 ± 0.61^a^	22.05 ± 1.35^b^	13.17 ± 0.65^c^
Lymphocytes %	9.05 ± 0.35^a^	7.55 ± 0.35^b^	3.88 ± 0.33^c^
Epithelial cells %	0.83 ± 0.18^a^	2.17 ± 0.40^b^	2.39 ± 0.50^c^
N:M ratio	0.27 ± 0.01^a^	4.2 ± 0.44^b^	6.37 ± 0.31^c^
*Staphylococcus aureus* (log CFU/mL)	ND	5.60 ± 0.02^a^	6.54 ± 0.01^b^
*Escherichia coli* (log CFU/mL)	ND	3.83 ± 0.02^a^	4.31 ± 0.05^b^
*Streptococcus agalactiae*	ND	+	+

Values are expressed as mean ± SE. Values within a row with different superscript letters differ (p < 0.05) between groups.

SCC, somatic cell count; CFU, colony-forming unit; ND, not detected; +, haemolysis positive; DLC, differential leukocyte count; N:M, neutrophil:macrophage ratio; H, healthy; SCM, subclinical mastitis; CM, clinical mastitis; SNF, solid non-fat.

### Mastitis-causing pathogens identified in milk samples

The quarter milk samples that were tested and found negative for bacterial culture were categorized into the H group. Conversely, the causative pathogens responsible for subclinical and clinical mastitis cases were identified as *Staphylococcus aureus*, *Escherichia coli*, and *Streptococcus agalactiae* (**Table 1**).

### RNA isolation, library preparation, and quality control

The extracted RNA passed quality and quantity assessment with optimum yield and concentration suitable for Illumina Library preparations. All the samples had RIN ≥ 7.0, which were considered good and were taken for library preparation. The quantitative and qualitative results are shown in **Table 2**. The Illumina libraries showed an average fragment size of ~382 bp with sufficient concentration to obtain the desired amount of sequencing data. The descriptions of the libraries are presented in **Table 3**.

**Table 2:** RNA concentration and purity of milk somatic cells estimated in Sahiwal cows.

S.No.	Sample	NanoDrop QC					Qubit QC			Sample quality control			
		ng/µL	260/280	260/230	Volume (µL)	Yield (ng)	Qubit Conc. (ng/µL)	Volume (µL)	Yield (ng)	ND Purity ratios	Qubit yield	Tape RNA integrity	Tape #RIN
1.	H1	466.8	1.74	0.2	18	8,402.4	12	18	216	Admissible	Admissible	Optimal	7.3
2.	H2	37.8	1.79	0.07	18	680.4	10.9	18	196.2	Admissible	Admissible	Optimal	7
3.	H3	320.5	2.03	0.64	18	5,769	14.8	18	266.4	Admissible	Admissible	Optimal	8.9
4.	SCM1	591	1.79	0.28	20	11,820	94.5	20	1,890	Admissible	Optimal	Optimal	8.1
5.	SCM2	598.3	1.81	0.27	20	11,966	136	20	2,720	Admissible	Optimal	Optimal	9.1
6.	SCM3	430.3	1.7	0.24	18	7,745.4	96	18	1,728	Admissible	Optimal	Optimal	8.6
7.	CM1	600.9	1.95	0.78	18	10,816.2	481.6	18	8,668.8	Admissible	Optimal	Optimal	8.4
8.	CM2	1,055.2	1.81	0.42	18	18,993.6	288	18	5,184	Admissible	Optimal	Optimal	9.4
9.	CM3	283.6	1.98	0.53	18	5,104.8	29.12	18	524.16	Admissible	Optimal	Optimal	8.4

QC, quality control; RIN, RNA integrity.

**Table 3:** Description of the libraries.

S. No.	Sample ID	Qubit concentration (ng/μL)	Vol (μL)	Yield (ng)	Barcode 1	Index sequence 1	Barcode 2	Index sequence 2
1.	H1	41.4	10	414	S766	TATGGCAC	S555	TTGCGAGA
2.	H2	45.0	10	450	S711	GAATCACC	S560	GAACGAAG
3.	H3	1.90	10	19	S763	CAAGGTAC	S505	CGTATCTC
4.	SCM1	18.6	10	186	S771	GTCAGTCA	S531	CGGCATTA
5.	SCM2	26.6	10	266	S779	CCTTCCAT	S589	CACGCAAT
6.	SCM3	6.19	10	61.9	S797	AGACCTTG	S501	TTACGTGC
7.	CM1	10.9	10	109	S739	CTTACAGC	S503	TGGTGAAG
8.	CM2	42.0	10	420	S737	TACCTGCA	S576	GGACATCA
9.	CM3	25.4	10	254	S728	AGACGCTA	S582	GGTGTACA

### Illumina sequencing

The data obtained from the sequencing run were de-multiplexed by Bcl2fastq software v2.20 using the unique dual barcode sequences, and Fastq files were generated. The sequencing quality was assessed using the FastQC v0.11.8 software. The adapter sequences were trimmed, and bases above q30 were considered. Low-quality bases were filtered off during read pre-processing and used for downstream analysis. The raw data sequencing quality was assessed. The adapter sequences were trimmed, and low-quality bases were filtered off during read pre-processing. The reads with a Phred score > q30 were used in downstream analysis.

### mRNA sequencing statistics

Using Illumina sequencing technology, for nine samples, an average of 23.64 million paired-end raw data were generated, where an average of 23.09 million paired-end reads were retained as high-quality (>q30) data. Nearly 97.41% of total reads were retained as high-quality (>q30) data and are presented in **Table 4**.

**Table 4:** Illumina paired-end read statistics.

S. No.	Sample	Raw reads	Processed reads	% of High-quality data	% Alignment (*Bos taurus*)
1.	H1	25,673,262	25,190,889	98.12%	87.94%
2.	H2	27,002,087	26,432,292	97.89%	90.24%
3.	H3	11,815,226	11,012,474	93.21%	82.83%
4.	SCM1	25,098,499	24,658,120	98.25%	95.15%
5.	SCM2	24,944,794	24,538,464	98.37%	94.18%
6.	SCM3	17,762,468	17,342,488	97.64%	93.41%
7.	CM1	27,159,136	26,618,751	98.01%	51.77%
8.	CM2	24,391,974	24,035,866	98.54%	80.72%
9.	CM3	28,913,846	27,956,326	96.69%	77.97%

### Transcriptomic profiling of milk SCs

**Figure 2:**
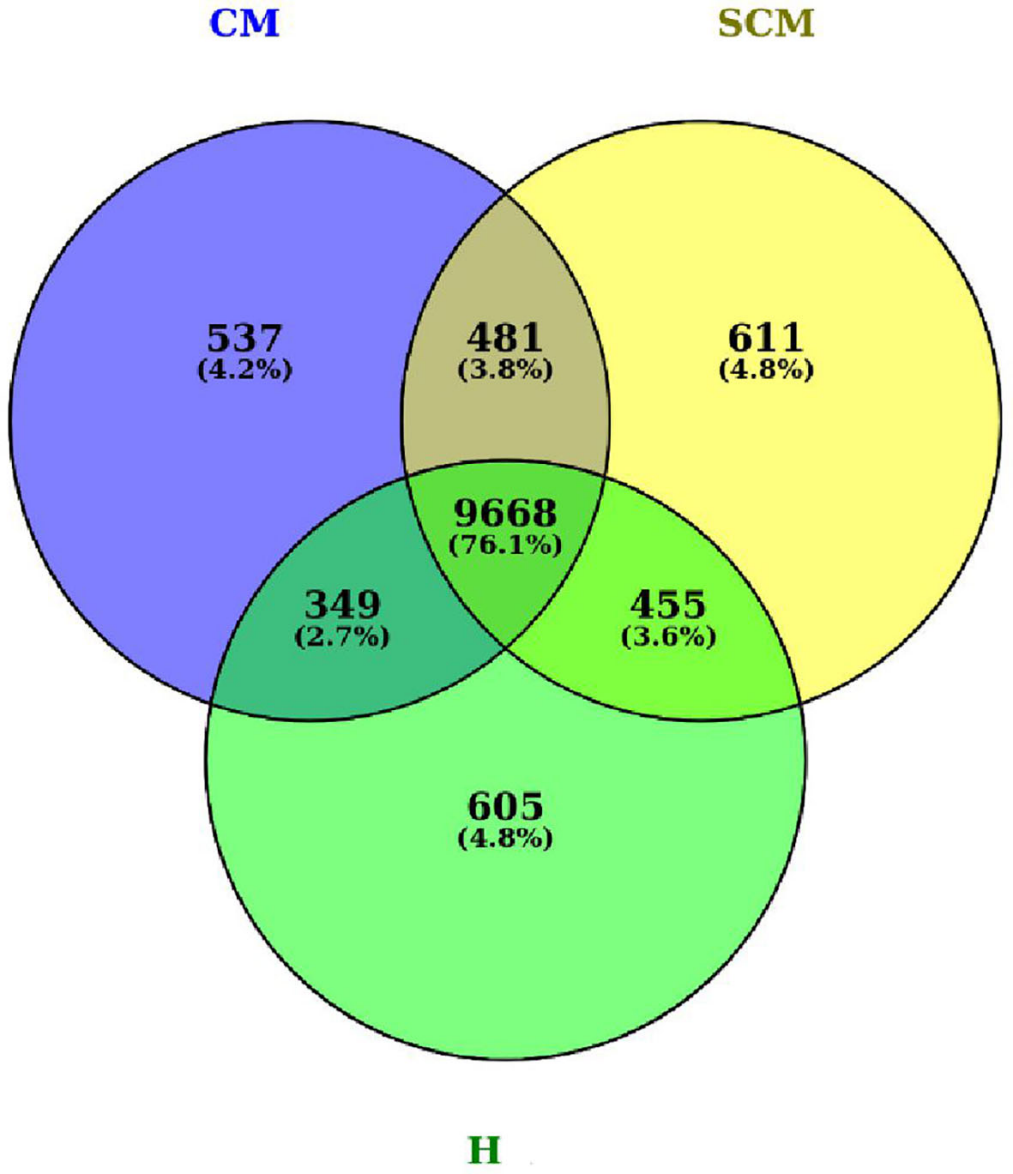
Venn diagrams representing the total identified genes (mRNA transcript) in milk somatic cells of CM, SCM, and H groups of cows. H, healthy; SCM, subclinical mastitis; CM, clinical mastitis.

On exploratory data analysis, 481, 349, and 455 genes were common to CM vs. SCM, CM vs. H, and SCM vs. H, respectively. Additionally, 537, 611, and 605 unique genes were exclusively identified in the CM, SCM, and H groups, respectively. The Venn diagram visually portrays the distinct proteins identified under the conditions of H, SCM, and CM, showcasing their overlap and uniqueness in **Figure 2**.

### Differentially expressed genes of milk SCs during mastitis

**Figure 3:**
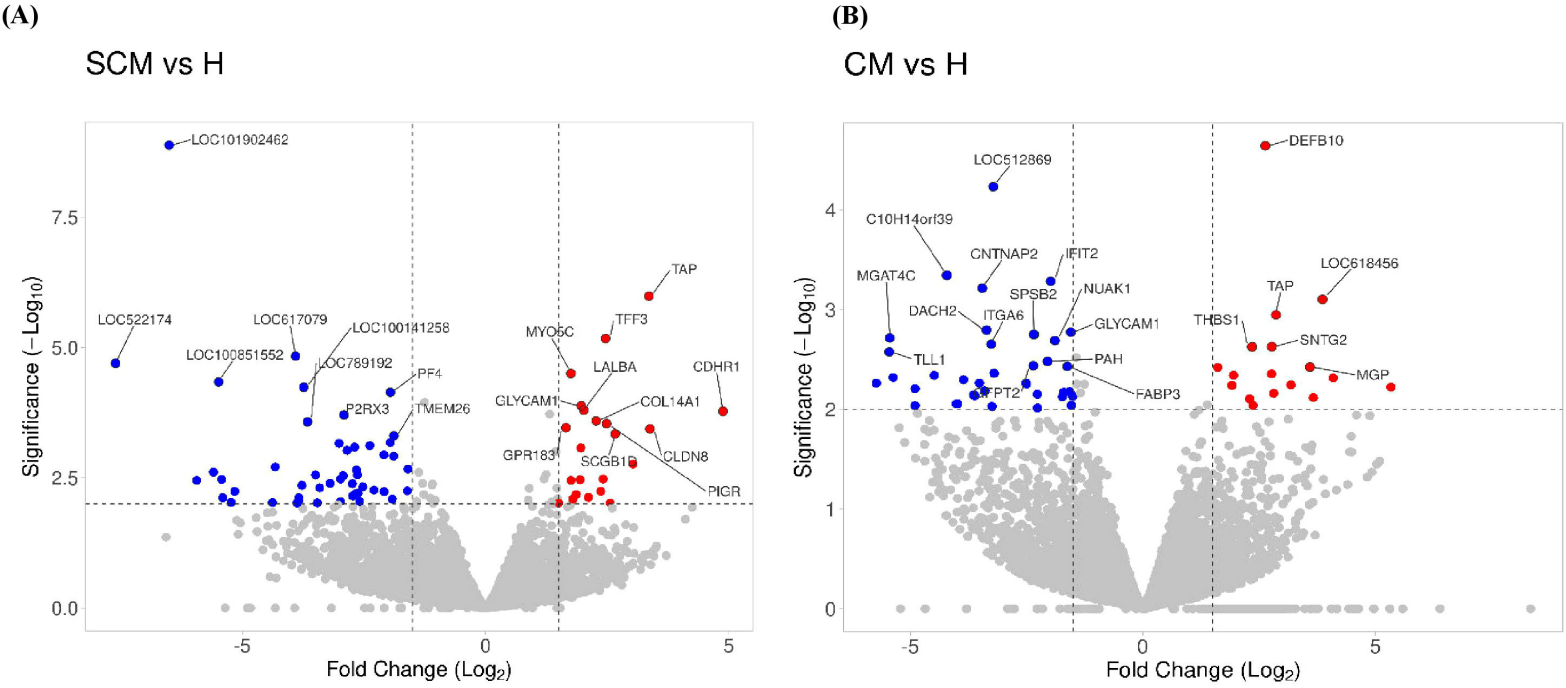
Volcano plot showing the gene expression profiles: **(A)** SCM vs. H, and **(B)** CM vs. H. The reads were quantified as reads per kilobase million (RPKM) and normalized with trimmed mean and Z-score across all samples. The fold changes were Log_2_ transformed. The p-values were transformed with −Log_10_. Cut-off points were p < 0.05 and absolute fold change ≥1 and ≤−1. The blue dots indicate significantly downregulated genes; the red dots indicate significantly upregulated genes. DEGs, differentially expressed genes; H, healthy; SCM, subclinical mastitis; CM, clinical mastitis.

**Figure 4:**
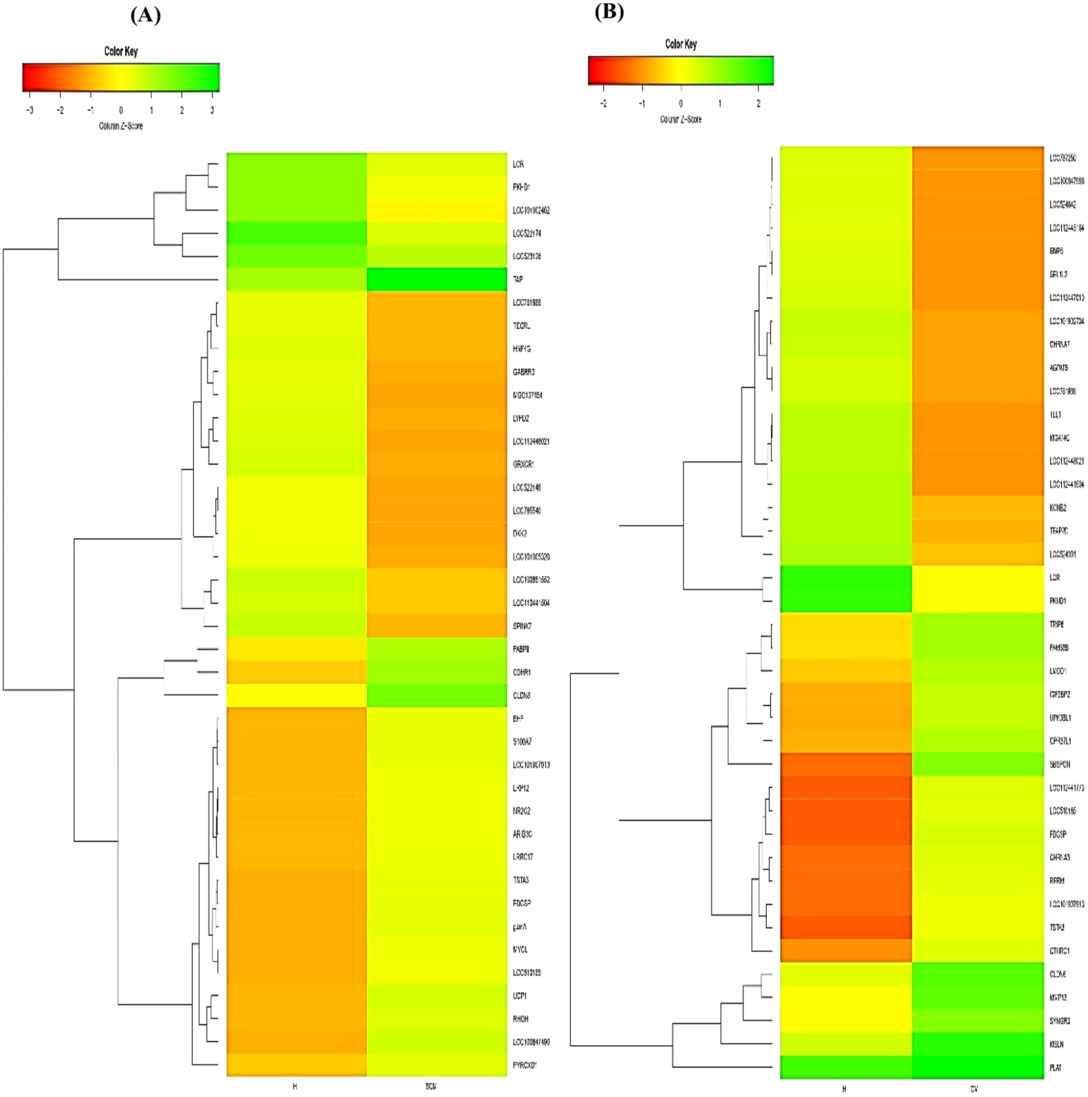
Heatmap of all milk somatic cell DEGs showing differential expression among the biological replicates of **(A)** SCM vs. H and **(B)** CM vs. H groups. The red colour represents upregulated expression of the specific significant (p < 0.05) gene, while green colour represents the downregulation of specific genes. The dendrogram on the y-axis of the heatmap represents the relatedness or how similar 2/more transcripts are to each other. The transcripts that cluster together are similar to each other based on normalized values. DEGs, differentially expressed genes; H, healthy; SCM, subclinical mastitis; CM, clinical mastitis.

**Figure 5:**
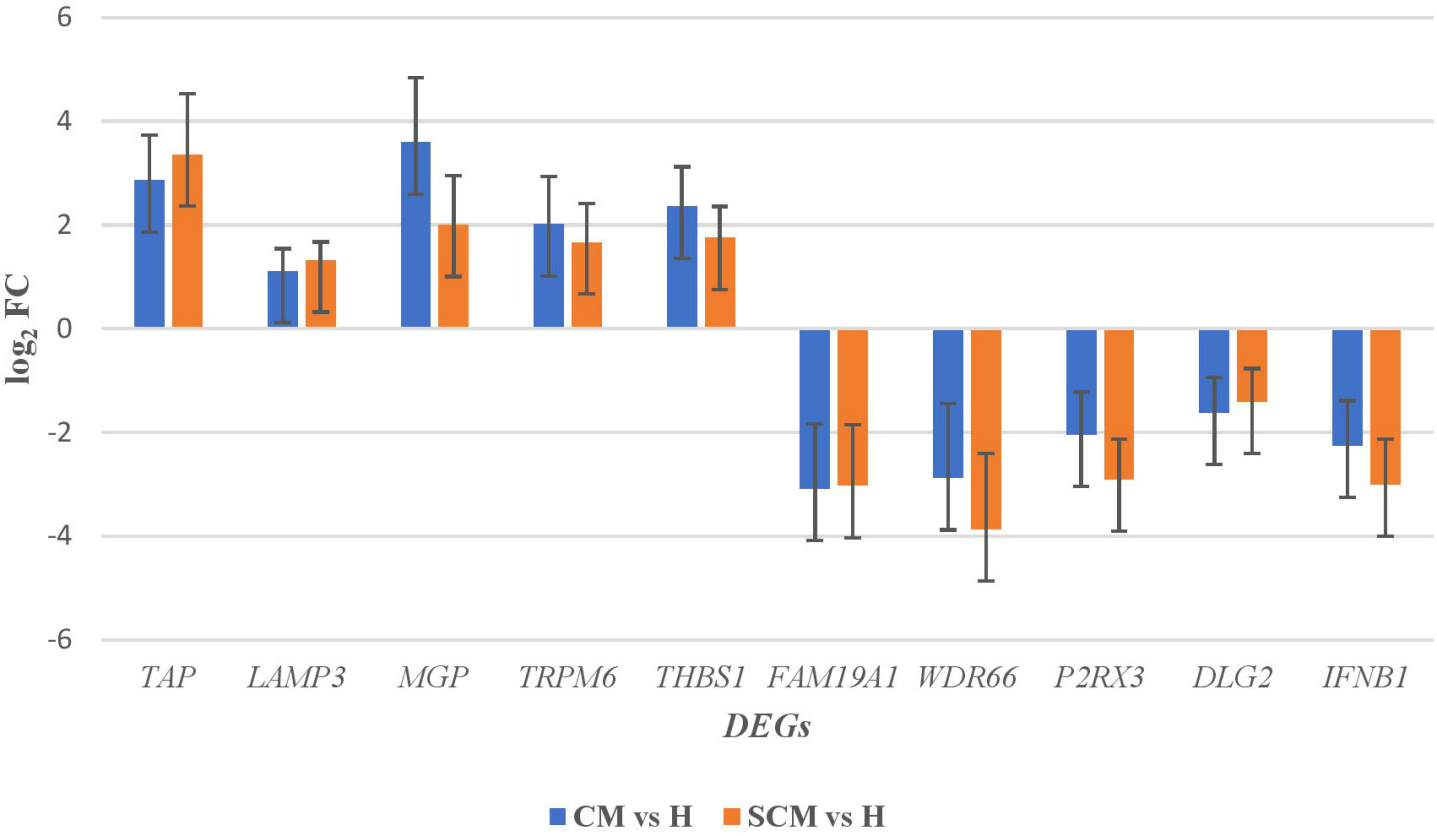
The top significant (p < 0.05) up- and downregulated DEGs in milk somatic cells along with its fold change. DEGs, differentially expressed genes.

Out of the specific DEGs, 83 and 76 DEGs were identified and showed significant upregulation (p < 0.05, Log_2_FC ≥ 1) in the CM vs. H and SCM vs. H groups, respectively. However, the significantly downregulated DEGs (p < 0.05, Log_2_FC ≤ −1) in the CM vs. H and SCM vs. H groups were 157 and 192, respectively. The top significant up- and downregulated DEGs identified in each condition are represented in the volcano plot (**Figure 3**). The alterations in gene expression are visually depicted in the heatmap, showcasing the hierarchical clustering of significantly expressed milk somatic cell genes, indicating changes in the expression level during different transitions of the disease in the SCM and CM groups when compared to the H group. The bar colour represents a logarithmic scale from −1 to 2 significant proteins (**Figure 4**). The DEGs that were top significantly (p < 0.05) upregulated in the CM and SCM groups played roles in immune-mediated functions during mastitis. Correspondingly, the significant DEGs downregulated during mastitis were mostly involved in the metabolic processes represented in **Figure 5**. The upregulated DEGs were immune-related genes that showed higher expression in both CM and SCM compared to healthy cows (H). Notably, transporter associated with antigen processing (TAP; involved in antigen processing) exhibited the highest upregulation in SCM (Log_2_FC = 3.361, p = 0.00) and CM (Log_2_FC = 2.864, p = 0.001), indicating its role in immune activation. Similarly, matrix Gla protein (MGP), known for its role in tissue remodelling, showed strong upregulation in CM (Log_2_FC = 3.596, p = 0.003) and SCM (Log_2_FC = 2.005, p = 0.035). Other upregulated genes, such as lysosome-associated membrane protein 3 (LAMP3) in CM (Log_2_FC = 1.11, p = 0.0) and SCM (Log_2_FC = 1.32, p = 0.000), transient receptor potential melastatin 6 (TRPM6) in CM (Log_2_FC = 2.01, p = 0.03) and SCM (Log_2_FC = 1.665, p = 0.03), and thrombospondin 1 (THBS1) in CM (Log_2_FC = 2.35, p = 0.002) and SCM (Log_2_FC = 1.76, p = 0.003), play critical roles in immune signalling, macrophage activation, and extracellular matrix organization. The most significantly downregulated genes in the mastitis groups were involved in immune regulation and epithelial integrity. Family with sequence similarity 19 (FAM19A1) showed the strongest suppression (Log_2_FC = −3.079, p = 0.01 in CM and −3.030 in SCM, p = 0.01), followed by WD repeat domain containing domain 66 (WDR66). The greater downregulation of WDR66 in SCM (Log_2_FC = −3.871, p = 0.01) compared to CM (Log_2_FC = −2.882, p = 0.04) suggests that distinct metabolic and immune adaptations were higher in early-stage infections. Purinergic receptor (P2RX3) in CM (Log_2_FC = −2.044, p = 0.01) and SCM (Log_2_FC = −2.90, p = 0.00), disks large homolog 2 (DLG2) in CM (Log_2_FC = −1.617, p = 0.02) and SCM (Log_2_FC = −1.41, p = 0.03), and interferon beta (IFNβ1) in CM (Log_2_FC = −2.26, p = 0.01) and SCM (Log_2_FC = −3.00, p = 0.001) indicated a potential weakening of immune modulation and barrier function.

### Gene network analysis of DEGs

**Figure 6:**
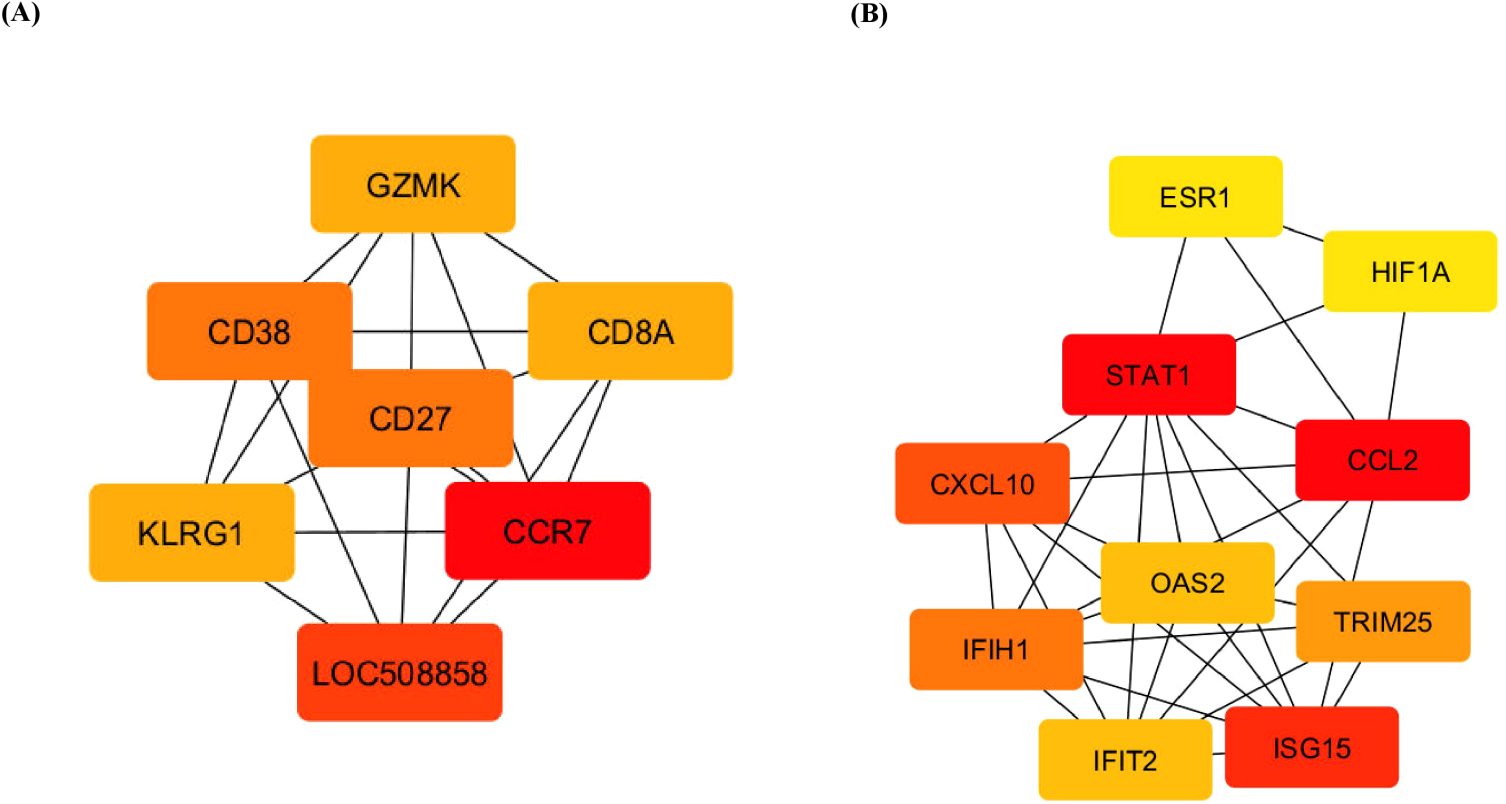
Top 10 hub genes involved in DEGs. **(A)** Upregulated and **(B)** downregulated differentially expressed genes using cytoHubba according to degree. Higher ranking is represented by a redder colour. DEGs, differentially expressed genes.

The identification of hub genes is crucial for understanding the central regulatory mechanisms in complex biological networks. Using the CytoHubba plugin in Cytoscape, the DEGs were analysed to identify hub genes. CytoHubba ranks nodes based on various topological algorithms, highlighting key genes that play central roles in the network. This visual distinction helps researchers easily identify and focus on the most influential genes within the network that play crucial roles in biological processes. The hub genes *CCR7*, *CD27*, *CD38*, *CD8A*, *GZMK*, and *KLRG1* were identified for the upregulated DEGs across the groups, whereas genes such as *STAT1*, *CCL2*, *CXCL10*, *ISG15*, *IFIH1*, and *IFIT2* were the observed hub genes expressed by the downregulated DEGs across the groups, which are presented in **Figure 6**. Assessments of these gene interactions confirm our hypothesis that the milk SCs carry quantitative signatures of genes responsible for host immune defence against naturally infected mastitis.

### Gene Ontology functional analysis of DEGs

Gene Ontology (GO) analysis offers a comprehensive understanding of the biological significance of DEGs by categorizing them into biological processes (BPs), cellular components (CCs), and molecular functions (MFs). Using the DAVID, this analysis identifies key pathways and molecular mechanisms involved in the transcriptomic data of milk SCs during mastitis, revealing critical insights into immune response, inflammation, and other affected biological processes. In the CM vs. H cows, these upregulated DEGs were linked to positive chemotaxis and the regulation of endothelial cell apoptosis, with CCs highlighting extracellular regions and MFs focusing on CCR6 chemokine receptor binding and calcium ion binding. For SCM vs. H, BPs involved T-cell chemotaxis and negative regulation of interleukin-12 production, with CCs including extracellular space and receptor complexes, and MFs related to transmembrane signalling receptor activity (**Table 5**). Downregulated DEGs in CM vs. H were linked to chemokine-mediated signalling and immune response regulation, with MFs involving type I interferon receptor binding. In SCM vs. H, downregulated DEGs were associated with BPs such as neutrophil chemotaxis, with corresponding CCs and MFs focusing on extracellular space and CXCR chemokine receptor binding (**Table 6**).

**Table 5:** Gene Ontology terms on significant (p < 0.05) upregulated DEGs (Log_2_FC ≥ 1) in milk somatic cells.

Category	Term	Count	%	p-Value	Genes
*CM vs. H*					
GOTERM_BP	GO:0050918~positive chemotaxis	3	3.896	0.004	TAP, DEFB7, DEFB10
	GO:2000353~positive regulation of endothelial cell apoptotic process	2	2.597	0.032	PRKCI, THBS1
	GO:0060326~cell chemotaxis	3	3.896	0.033	TAP, DEFB7, DEFB10
GOTERM_MF	GO:0031731~CCR6 chemokine receptor binding	3	3.896	0.006	TAP, DEFB7, DEFB10
	GO:0005509~calcium ion binding	7	9.091	0.010	NOTCH3, SPATA21, MGP, CELSR1, MYL9, THBS1, FBN1
	GO:0042056~chemoattractant activity	3	3.896	0.011	TAP, DEFB7, DEFB10
*SCM vs. H*					
GOTERM_BP	GO:0006954~inflammatory response	5	6.76	0.010	CCL22, TSPAN2, CCR7, THBS1, CCL26
	GO:0006955~immune response	5	6.76	0.014	TGFBR3, SERPINB9, CCR7, THBS1, BOLA-DOB
	GO:0010818~T-cell chemotaxis	2	2.70	0.031	GPR183, CCL26
	GO:0016477~cell migration	4	5.41	0.034	TGFBR3, SDC2, TSPAN11, THBS1
	GO:0060326~cell chemotaxis	3	4.05	0.037	TAP, DEFB10, CCR7
	GO:0032695~negative regulation of interleukin-12 production	2	2.70	0.038	CCR7, THBS1
	GO:0050921~positive regulation of chemotaxis	2	2.70	0.045	THBS1, CCL26
GOTERM_MF	GO:0004888~transmembrane signalling receptor activity	3	4.054054	0.043	FCRLA, PIGR, CD27

DEGs, differentially expressed genes.

**Table 6:** Gene Ontology terms on significant (p < 0.05) downregulated DEGs (Log_2_FC ≤ −1) in milk somatic cells.

Category	Term	Count	%	p-Value	Genes
*CM vs. H*					
	GO:0032600~chemokine receptor transport out of membrane raft	2	1.351	0.014	CD24
	GO:0007155~cell adhesion	7	4.730	0.016	DSCAM, PCDH15, NRXN3, ITGA6, CD24, FOLR1
	GO:0032728~positive regulation of interferon-beta production	3	2.027	0.020	IFIH1, OAS2, ISG15
	GO:0001959~regulation of cytokine-mediated signalling pathway	2	1.351	0.027	CD24
	GO:0002323~natural killer cell activation involved in immune response	3	2.027	0.033	LOC525550, IFNB1, LOC517016
	GO:0002286~T-cell activation involved in immune response	3	2.027	0.038	LOC525550, IFNB1, LOC517016
	GO:0001775~cell activation	2	1.351	0.047	CD24
GOTERM_MF	GO:0005132~type I interferon receptor binding	4	2.703	0.002	LOC525550, IFNB1, LOC517016, LOC112441471
	GO:0005125~cytokine activity	5	3.378	0.028	LOC525550, IFNB1, LOC517016, LOC112441471, BMP5
*SCM vs. H*					
GOTERM_BP	GO:0006952~defence response	4	2.186	0.001	CXCL10, STAT1, PPBP, PF4
	GO:0030593~neutrophil chemotaxis	5	2.732	0.001	CXCL10, CCL8, ITGA1, PPBP, PF4
	GO:0070098~chemokine-mediated signalling pathway	4	2.186	0.006	CXCL10, CCL8, PPBP, PF4
	GO:0031640~killing of cells of other organisms	3	1.639	0.020	LOC781146, LYZ3, LAP
	GO:0007157~heterophilic cell–cell adhesion via plasma membrane cell adhesion molecules	3	1.639	0.026	TENM1, GRID2, TENM3
GOTERM_MF	GO:0045236~CXCR chemokine receptor binding	3	1.639	0.003	CXCL10, PPBP, PF4
	GO:0008009~chemokine activity	4	2.186	0.003	CXCL10, CCL8, PPBP, PF4
	GO:0005125~cytokine activity	6	3.279	0.011	LOC525550, IFNA16, IFNB1, IL12A, LOC112441471, BMP7
	GO:0048248~CXCR3 chemokine receptor binding	2	1.093	0.027	CXCL10, PF4

DEGs, differentially expressed genes; CM, clinical mastitis; H, healthy; SCM, subclinical mastitis.

### KEGG pathway enrichment analysis of DEGs during mastitis

**Figure 7:**
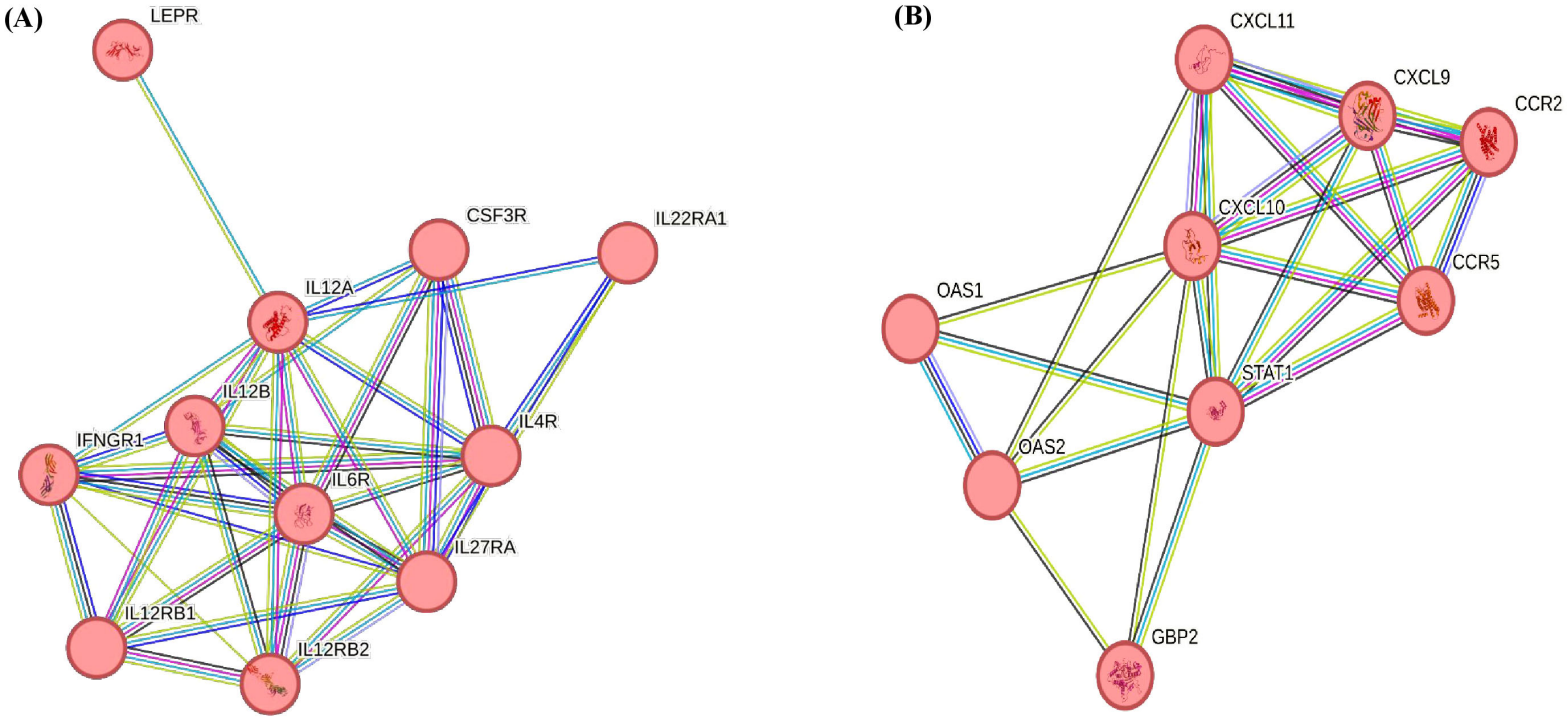
**(A)** Cytokine receptor activity obtained through MCL clustering: no. of nodes, 11; no. of edges, 36; avg. local clustering co-efficient 0.879; interaction value, 3.6e−10. **(B)** Chemokine receptor activity obtained through MCL clustering: no. of nodes, 9; no. of edges, 24; avg. local clustering co-efficient 0.849; interaction value, <1.0e−16.

The KEGG pathways associated with upregulated DEGs (p < 0.05, Log_2_FC ≥ 1) in CM vs. H included phagosome, IL-17 signalling pathway, and Th1 and Th2 cell differentiation. For SCM vs. H, the key pathways were cell adhesion molecules (**Table 7**). However, in downregulated DEGs (p < 0.05, Log_2_FC ≤ −1) in SCM vs. H, highlighted pathways included RIG-I-like receptor signalling pathway, natural killer cell-mediated cytotoxicity, NOD-like receptor signalling pathway, tumour necrosis factor (TNF) signalling pathway, toll-like receptor signalling pathway, and cytokine–cytokine receptor interaction (**Table 8**). The cytokine–cytokine interaction and chemokine–chemokine interaction through MCL clustering and respective no. of nodes, no. of edges, avg. local clustering coefficient, and interaction values obtained are depicted in **Figure 7**. However, the comparative analysis with proteomic data of milk SCs identified that the downregulated DEPs in SCM vs. H were also involved in chemokine signalling. The downregulation of the chemokine CXCL10, CCL2, and CCL8 genes may therefore indicate weakened antibacterial responses, impacting the gland’s ability to clear infections effectively.

**Table 7:** KEGG pathway enrichment analysis on significant (p < 0.05) high-abundance DEGs (Log_2_FC ≥ 1) in milk somatic cells.

Category	Term	Count	%	p-Value	Genes
*CM vs. H*	bta04145: Phagosome	4	5.195	0.017	C3, CTSV, THBS1, BOLA-DOB
	bta04657:IL-17 signalling pathway	3	3.896	0.036	TAP, CCL17, LOC100847175
	bta04658: Th1 and Th2 cell differentiation	3	3.896	0.039	NOTCH3, LOC510185, BOLA-DOB
*SCM vs. H*	bta04514: Cell adhesion molecules	7	9.459459	5.32E−05	CD8A, SDC2, CLDN8, GLYCAM1, MPZL1, JAM2, BOLA-DOB

KEGG, Kyoto Encyclopedia of Genes and Genomes; DEGs, differentially expressed genes; CM, clinical mastitis; H, healthy; SCM, subclinical mastitis.

**Table 8:** KEGG pathway enrichment analysis on significant (p < 0.05) low-abundance DEGs (Log_2_FC ≤ −1) in milk somatic cells.

Category	Term	Count	%	p-Value	Genes
*CM vs. H*	bta04622: RIG-I-like receptor signalling pathway	7	4.730	0.000	IFIH1, LOC525550, IFNB1, LOC517016, TRIM25, ISG15, LOC112441471
	bta04650: Natural killer cell-mediated cytotoxicity	8	5.405	0.000	LOC525550, IFNB1, LOC517016, LOC112445512, PIK3R3, LOC104968444, LOC112441471, ULBP13
	bta04621: NOD-like receptor signalling pathway	8	5.405	0.001	OAS1X, OAS1Y, LOC525550, IFNB1, OAS2, LOC517016, CCL2, LOC112441471
	bta04668: TNF signalling pathway	6	4.054	0.003	LOC525550, IFNB1, LOC517016, PIK3R3, CCL2, LOC112441471
*SCM vs. H*	bta04622: RIG-I-like receptor signalling pathway	7	3.825	5.45E−04	LOC525550, CXCL10, IFNA16, IFNB1, IL12A, ISG15, LOC112441471
	bta04620: Toll-like receptor signalling pathway	7	3.825	9.98E−04	LOC525550, CXCL10, IFNA16, IFNB1, STAT1, IL12A, LOC112441471
	bta04060: cytokine–cytokine receptor interaction	10	5.464	0.005	LOC525550, CXCL10, CCL8, IFNA16, IFNB1, IL12A, PPBP, LOC112441471, BMP7, PF4
	bta04062: Chemokine signalling pathway	7	3.825	0.010	CXCL10, CCL8, STAT1, ADCY2, PPBP, GNG11, PF4
	bta04621: NOD-like receptor signalling pathway	6	3.279	0.038	LOC525550, IFNA16, IFNB1, STAT1, LAP, LOC112441471

KEGG, Kyoto Encyclopedia of Genes and Genomes; DEGs, differentially expressed genes; CM, clinical mastitis; H, healthy; SCM, subclinical mastitis.

### Validation of chemokine DEGs of RNA-seq data by qRT-PCR

The relative mRNA expression levels of CCR7 and CCL22 were significantly (p < 0.05) higher in the SCM group compared to the H group; CXCL10, CCL8, and mRNA expression levels were significantly (p < 0.05) lower in the SCM group compared to the H group; CCL17 was significantly higher in the CM group compared to the H group; and a significant reduction of CCL2 occurred in the CM group compared to the H group. The qRT-PCR results showed that the expressions of the selected DEGs were consistent with the changing trends from RNA-seq data, but there were differences in fold changes between RNA-seq and qRT-PCR, thereby validating the RNA-seq results presented in **Table 9**, which shows the relative gene expression using qRT-PCR (2^−ΔΔCT^ method) (26).

**Table 9:** Comparison of Log_2_ fold change expression values of significant (p < 0.05) chemokine regulated in mastitis conditions through RNA-seq and qPCR values.

Gene name	Description	RNA-seq values	qPCR values	Functions
		Log_2_FC	Log_2_FC	
CCR7	C-C motif receptor 7	1.440	5.229	Regulates homing of immune cells​​
CCL22	C-C motif chemokine ligand 22	1.561	8.693	Induce chemotaxis in T-helper cells
CXCL10	C-X-C motif chemokine ligand 10	−1.885	0.227	Chemotaxis, induction of apoptosis
CCL8	C-C motif chemokine ligand 8	−1.481	0.389	Neutrophil chemotaxis, chemokine-mediated activity
CCL 17	C-C chemokine ligand 17	1.418	3.398	CCL17 was the first CC chemokine identified that interacted with T cells with high affinity
CCL2	C-C motif chemokine ligand 2	−0.941	0.066	CCL2 is a novel target for improving the quantity and quality of milk from cows through stimulation of proliferation on mammary epithelial cells.

## Discussion

### Milk SCCs and leukocyte profiles during mastitis

Milk SCCs and DLCs provide valuable insights into the mammary glands’ inflammatory responses and cellular dynamics during mastitis. The elevation in SCCs, attributed to immune cell migration to the infection site, reflects mammary inflammation. A significant variation in SCCs (p < 0.05) was observed among different groups. Additionally, DLC analysis showed shifts in primary cell populations between healthy and mastitis-affected groups. Milk macrophages are more abundant in healthy mammary glands, with neutrophils constituting approximately 20%. However, in infected glands, neutrophils increase to over 50%, while macrophages (%) decrease, indicating infection (2, 27, 28).

Microbiological analysis of milk, combined with SCC assessment, offers crucial insights into the prevalence of specific mastitis-causing pathogens and the level of inflammation in dairy herds (12). The primary pathogens in this region include *S. aureus*, *E. coli*, and *S. agalactiae*, which often cause SCM and may progress to CM, proving challenging to eliminate from a herd. Natural infections in cows typically progress from subclinical to clinical mastitis, characterized by the activation of the mammary gland and a substantial neutrophil influx in milk, indicating that upregulated immunity-related genes are key to host defence.

### Transcriptomic analysis of gene expression changes

In this study, transcriptomic analysis of gene expression changes during mastitis revealed that 23.09 million paired-end reads (97.41%) were retained as high-quality data compared to the reference genome. Significant genes were screened based on a fold change >1 for upregulated and <−1 for downregulated mRNA expression with a p-value <0.05 in the mastitis groups compared to healthy counterparts. The significantly upregulated genes associated with immune mechanisms during the onset of inflammation include TAP, involved in MHC class I processing and presentation (29) (30); LAMP3 is associated with genes involved in T-cell activation, macrophage maturation, and cytokine and chemokine release, enhancing dendritic cell maturation and thus playing a vital role in orchestrating inflammatory responses during infections (31). MGP is a vitamin K-dependent protein primarily known for its role in regulating vascular calcification and bone metabolism and may also influence immune responses and tissue remodelling in inflammatory conditions (32). TRPM6 is involved in magnesium homeostasis and is expressed in mammary epithelial cells, where it is crucial for cellular functions and may impact immune cell activity during inflammatory responses (33, 34). CCR7 regulates immune cell homing during subclinical infections (35), THBS1 is a cell adhesion molecule involved in extracellular matrix organization and inflammation (36), and PIGR mediates the transcytosis of IgA and IgM (37, 38). Conversely, the top downregulated genes in both clinical and subclinical mastitis groups, such as FAM19A1, WDR66, P2RX3, DLG2, and IFNB1, were associated with immune regulation and mammary epithelial integrity, underscoring their roles in modulating inflammatory responses and maintaining mammary barrier function (39–43). Additionally, the significantly downregulated DEGs in the clinical mastitis group compared to the subclinical group were primarily involved in metabolic processes, including fatty acid metabolism, with genes such as SLC24A2 (44), SCD (45), FABP3 (46), GLYCAM1 (47), and SCGB1D (8), highlighting their potential roles in mastitis resistance and the metabolic adjustments during infection. These findings provide insights into mastitis pathogenesis’s complex genetic mechanisms and offer potential avenues for future research.

### Transcriptomic analysis of hub genes and pathway signalling

The hub genes identified among the upregulated DEGs included CCR7, CD27, CD38, CD8A, GZMK, and KLRG1. Conversely, the downregulated DEGs were associated with genes such as STAT1, CCL2, CXCL10, ISG15, IFIH1, and IFIT2. CCR7, THBS1, PIGR, and cluster of differentiation molecules such as CD 27, a member of the TNF receptor superfamily, and is expressed in T, B, and NK cells (48), CD38, a cell activation marker for T cells, NK cells, B cells, and dendritic cells (49), and CD8A (alpha chain), a T-cell receptor signalling pathway (48). The killer cell lectin-like receptor subfamily G member 1 (KLGR1) in leucocyte regulation in both the innate and adaptive immune systems, CD8+ T and NK cells, and Granzyme K (GZMK) promotes TLR4 signalling during the antimicrobial innate immune response (50).

### Potential DEG biomarkers associated with cytokine–chemokine immune response

*S. aureus* was the predominant pathogen in our study; the absence of significant CXCL8 expression aligns with previous reports, indicating a weaker neutrophil-driven response compared to infections caused by Gram-negative bacteria (51). Hence, it may be due to that reason that CXCL8 was not significantly expressed in our study. Instead of CXCL8-mediated neutrophil recruitment, we observed significant downregulation of other chemokines such as C-X-C motif chemokine ligand 10 (CXCL10; Log_2_FC = −1.885, p = 0.03), C-C motif chemokine ligand 8 (CCL8; Log_2_FC = −1.481, p = 0.03), and C-C motif chemokine ligand 2 (CCL2; Log_2_FC = −0.941, p = 0.04), suggesting a potential impairment in immune cell trafficking and pathogen clearance. Our transcriptomic analysis further confirmed these trends, showing a consistent downregulation of these chemokines at both the mRNA and qPCR validation levels.

Additionally, we examined the interferon signalling pathway, which plays a crucial role in regulating immune responses during mastitis. While IFN-λR1 (*Interferon Lambda Receptor 1*, Log_2_FC = 1.067, p = 0.03) significantly upregulated, indicating potential activation of antiviral and pro-inflammatory pathways, we observed a significant downregulation of IFN-β1 (*Interferon Beta 1*, Log_2_FC = −2.59, p = 0.001) and interferon-stimulated genes such as IFIT5 (*Interferon-Induced Protein with Tetratricopeptide Repeats 5*, Log_2_FC = −1.172, p = 0.002), IFIT2 (*Interferon-Induced Protein with Tetratricopeptide Repeats 2*, Log_2_FC = −1.577, p = 0.01), and IFI27 (*Interferon Alpha-Inducible Protein 27*, Log_2_FC = −2.28, p = 0.002), suggesting a suppressed type I interferon response. This could contribute to the decreased expression of CXCL10, a chemokine primarily induced by IFN-γ. Furthermore, the TNF superfamily genes, TNFSF13β (Log_2_FC = 2.453, p = 0.04) and TNFSF14 (Log_2_FC = 0.755, p = 0.02) were significantly upregulated, reinforcing an inflammatory response.

Regarding interleukin expression, IL18RAP (*Interleukin-18 Receptor Accessory Protein*, Log_2_FC = 0.800, p = 0.03) was significantly upregulated, which may contribute to immune cell activation and inflammation. Interestingly, while CCL8 (Log_2_FC = −1.481, p = 0.03) and CCL2 (C-C motif chemokine 2, Log_2_FC = −0.941, p = 0.04) were downregulated, other chemokines such as CCR7 (*C-C Chemokine Receptor 7*, Log_2_FC = 1.44, p = 0.01), CCL22 (C-C motif chemokine 22, Log_2_FC = 1.56, p = 0.01), and CCL17 (C-C chemokine ligand 17, Log_2_ FC = 1.418, p = 0.02) were upregulated, suggesting a shift towards an adaptive immune response rather than a robust innate immune activation. This differential expression pattern highlights the complex regulatory mechanisms in bovine mastitis and underscores the need for further investigations into immune evasion and potential immunomodulatory interventions.

In our study, the chemokines CCL17, CCL22, CCL8, CCL2, and CXCL10 were validated through qPCR as significant mediators in the immune response during bovine mastitis, particularly in regulating immune cell chemotaxis and activation. The upregulation of CCL17, the first CC chemokine discovered to bind T cells with high affinity, has been shown to play a critical role in attracting and activating T cells at sites of inflammation, enhancing the adaptive immune response. CCL22 further supports this process by inducing chemotaxis in T-helper cells, which is essential for amplifying T cell-mediated immunity in response to mastitis pathogens. However, the downregulation of CXCL10 is well known for inducing apoptosis, which may aid in controlling pathogen persistence by promoting the removal of infected or damaged cells (52). CCL8 promotes monocyte chemotaxis, essential innate immune system components, thus contributing to pathogen clearance and inflammation regulation (43). Their reduced expression may impair the trafficking of immune cells to the mammary gland, weakening the overall defence mechanism. An innovative approach to mitigate this issue could involve enhancing CXCL10 and CCL8 signalling through targeted therapies, such as gene editing or the use of specific agonists to restore their expression or function. This strategy could improve immune cell recruitment, potentially boosting the gland’s ability to respond to infections and enhancing overall resistance to mastitis. Recent studies suggest that gene-editing tools, such as CRISPR/Cas9, could be used to upregulate the expression of these chemokines, promoting effective immune responses in mastitis cows (53). Additionally, targeting CXCR3 with small-molecule agonists could amplify the chemotactic activity of CXCL10 (54), thus enhancing immune surveillance and pathogen clearance in the mammary gland.

These findings establish a foundation for innovative strategies to enhance mastitis resistance, including gene editing (CRISPR/Cas9) to restore chemokine expression, small-molecule agonists to strengthen immune responses, and selective breeding for genetic markers associated with robust immunity. The study also highlights the need for advanced breeding programs incorporating immune-genetic selection and supports vaccine development with adjuvants to enhance chemokine signalling, potentially reducing mastitis incidence and severity. These strategies hold promise for improving mastitis resistance, offering a novel approach to support immune function and enhance the defence against mastitis infection in dairy cattle.

## Conclusion

This is the first comprehensive transcriptomic analysis of gene expression changes during mastitis in Sahiwal breeds and has provided significant insights into the molecular mechanisms underlying the disease. High-quality data from 23.09 million paired-end reads revealed key upregulated genes such as TAP, LAMP3, MGP, RPM6, and THBS1, which are crucial for immune cell activation, tissue remodelling, and inflammatory responses, illustrating the complex immune dynamics in mastitis. Conversely, genes including FAM19A1, WDR66, P2RX3, DLG2, and IFNB1 were downregulated, reflecting disruptions in immune regulation and mammary epithelial integrity, with notable impacts on metabolic processes, particularly fatty acid metabolism, in clinical mastitis cases. Pathway analysis showed upregulated DEGs enriched in phagosome activity, IL-17 signalling, Th1 and Th2 cell differentiation, and cell adhesion molecules, while downregulated DEGs were linked to RIG-I-like receptor signalling, NK cell-mediated cytotoxicity, NOD-like receptor signalling, TNF signalling, Toll-like receptor signalling, cytokine–cytokine receptor interaction, and chemokine signalling pathways. Our study underscores the critical roles of chemokines CCL8, CCL2, and CXCL10 in immune cell recruitment and activation during bovine mastitis, where their downregulation may weaken mammary immune defence. Enhancing these key immune mediators through gene editing (CRISPR/Cas9), small-molecule agonists, and selective breeding for immune-genetic markers presents a promising strategy to improve mastitis resistance. Additionally, vaccine development incorporating adjuvants to restore chemokine signalling could further reduce mastitis incidence and severity. These findings not only advance our understanding of mastitis pathogenesis but also highlight the potential of chemokine biomarkers in refining therapeutic strategies, strengthening mastitis management, and enhancing dairy production outcomes.

## Data Availability

The data presented in the study are deposited in http://www.ncbi.nlm.nih.gov/bioproject/1214580 repository, accession number PRJNA1214580.
