# Supplementary material for: Cytokine interactions and chemokine dysregulations in mastitis immunopathogenesis: insights from transcriptomic profiling of milk somatic cells in tropical Sahiwal (Bos indicus) cows
Source: Front Immunol. 2025 Mar 24;16:1554341. doi: 10.3389/fimmu.2025.1554341 (PMC11973270; doi:10.3389/fimmu.2025.1554341)
Supplement: Supplementary file 1 [file Table1.docx]

**Supplementary Table 1.** Details of various primers used in the study.

| **S.No** | **Genes** | **Sequence (5′→3′)** | **Acc. no.** | **Size (bp)** |
| --- | --- | --- | --- | --- |
| 1. | CCR7 | F: AGAGAGTCATGGACCTGGGGAA  R: CCGTGGTGTTGTCTCCGAT | NM_001024930.3 | 123 |
| 2. | CCL17 | F: CAACAAAGCCATTGTGCTGGT  R: TGGGGGTTGGGGTAATGAAC | XM_002694792.5 | 192 |
| 3. | CCL22 | F: TCTGTTGCCGGGACTACATC  R: CTTCACAGTCAGCAAGACCAC | NM_001099162.2 | 110 |
| 4. | CCL8 | F: CAATCGCCAACTCTCAGGCT  R: GGGGTAGAAACTGAATCTGGCTG | NM_174007.1 | 137 |
| 5. | CXCL10 | F: CACGTGTCGAGATTATTGCC  R: GCTTCTCTCTGGTCCATCCT | NM_001046551.2 | 180 |
| 6. | CCL2 | F: TTATGTGCAGACCCCAAGCA  R: GGGAAAGCCGGAAGAACACA | NM_174006.2 | 137 |
| 7. | GAPDH | F: GGGTCATCATCTCTGCACCT  R: GGTCATAAGTCCCTCCACGA | NM_001034034.1 | 176 |
| 8. | ACTB | Forward: CCCTGGAGAAGAGCTACGAG  Reverse: GGATTCCATGCCCAGGAAGG | NM_173979.3 | 110 |
